# Epidemiological Characteristics on the Clustering Nature of COVID-19 in Qingdao City, 2020: A Descriptive Analysis

**DOI:** 10.1017/dmp.2020.59

**Published:** 2020-03-31

**Authors:** Jing Jia, Xiaowen Hu, Feng Yang, Xin Song, Liyan Dong, Jingfei Zhang, Fachun Jiang, Ruqin Gao

**Affiliations:** Municipal Centre of Disease Control and Prevention of Qingdao, Qingdao Institute of Prevention Medicine, Qingdao City, Shandong Province, P. R. China.

**Keywords:** cluster, COVID-19, epidemiology

## Abstract

**Objectives:**

As an emerging infectious disease, COVID-19 has involved many countries and regions. With the further development of the epidemic, the proportion of clusters has increased.

**Methods:**

In our study, we collected information on COVID-19 clusters in Qingdao City. The epidemiological characteristics and clinical manifestations were analyzed.

**Results:**

Eleven clusters of COVID-19 were reported in Qingdao City between January 29, and February 23, 2020, involving 44 confirmed cases, which accounted for 73.33% of all confirmed cases. From January 19 to February 2, 2020, the cases mainly concentrated in the district that had many designated hospitals. Patients aged 20-59 y old accounted for the largest proportion (68.18%) of cases; the male-to-female sex ratio was 0.52:1. Three cases were infected from exposure to confirmed cases. The average incubation period was 6.28 d. The median number of cases per cluster was 4, and the median duration time was 6 d. The median cumulative number of exposed persons was 53.

**Conclusion:**

More attention should be paid to the epidemic of clusters in prevention and control of COVID-19. In addition to isolating patients, it is essential to track, screen, and isolate those who have come in close contact with patients. Self-isolation is the key especially for healthy people in the epidemic area.

In December 2019, several cases of pneumonia of unknown etiology and not cause by any known respiratory virus infection were reported in Wuhan, Hubei province, China. The discovery and identification of the novel virus differs from the description of the 2 other zoonotic coronaviruses, severe acute respiratory syndrome coronavirus (SARS-CoV) and Middle East respiratory syndrome virus (MERS-CoV), introduced to humans in the 21st century.^1^ On February 11, 2020, this new infectious disease was officially named “COVID-19” by World Health Organization (WHO).^2^ The Coronavirus Study Group (CSG) of the International Committee on Taxonomy of Viruses formally designated the virus as severe acute respiratory syndrome coronavirus 2 (SARS-CoV-2).^3^ Given that the global challenges to public health attributed to the SARS-CoV-2 outbreak in China, WHO has declared the SARS-CoV-2 as a public health emergency of international concern.^4^

COVID-19 patients are the source of infection, and asymptomatic carriers are also infectious to other healthy persons. People are all generally susceptible to the virus that is transmitted mostly by means of droplets or contact, the transmission route achieved most easily.^5^ There has been plenty of evidence of sustained human-to-human transmission.^6^ As COVID-19 patients are increasing in number, so are cluster cases because of the highly infectious nature of this virus. Nearly 1000 cluster cases have been reported.^7^ Several control measures, such as containment and mitigation, are being implemented to contain the outbreak by the government,^8^ and the control and prevention of cluster events may be the key to a final victory.^9^ The next steps on the COVID-19 prevention and control should be focused on cluster outbreaks, including quarantining the source of infection, optimizing the management of close contacts, and preventing possible rebound of the epidemic after people return to work from the Chinese Spring Festival holiday.

The Health Commission of Qingdao, Shandong province, China, announced the first case of COVID-19 on January 21, 2020, and as of March 8, 2020, the number of confirmed cases in Qingdao has increased to 60.^10^ Cases of COVID-19 have been geographically dispersed, sporadic, and not linked to one another, especially in the early stage of COVID-19 infection. As the epidemic evolves, the number of confirmed cases from cluster outbreaks began to exceed sporadic cases, indicating a significant change in the source of exposure. Timely understanding of the characteristics of the COVID-19 clusters is the basis of targeting outbreak mitigation. In our study, we provide an analysis of data on the characteristics of cluster cases with confirmed COVID-19 throughout Qingdao, a port city, and hope to provide the basis for future prevention decisions of the current epidemic if similar dynamics apply elsewhere.

## METHODS

### Sources of Data

The data of cluster cases were obtained from the National Notifiable Disease Surveillance System. The demographics and epidemiological information were included in the investigation report. The epidemiological questionnaire was collected onto standardized forms through interviews of cases, asymptomatic infected persons, and close contacts. All epidemiologic information collected during field investigations, including exposure history, time-lines of events, close contact identification, and so on.

### Case Definitions

The criteria was as follows. A confirmed case was defined by positive respiratory specimens and clinical symptoms. A cluster of COVID-2019 was defined as 2 or more confirmed cases or asymptomatic cases found in a small area (such as home, office, class, workshop, and so on), with the possibility of human-to-human transmission or infection caused by joint exposure. The respiratory specimens were tested by real-time reverse-transcription-polymerase-chain-reaction (RT-PCR) assay for SARS-CoV-2 or a genetic sequence that matches SARS-CoV-2. Asymptomatic cases of COVID-19 were diagnosed based on positive viral nucleic acid test results but without any clinical symptoms. A close contact was defined as a person who did not take effective protection against a suspected or confirmed case 2 d before the onset of symptoms or an asymptomatic infected person 2 d before sampling.

### Laboratory Testing

Upper or lower respiratory tract specimens were sampled from patients. The samples were divided and tested in biosafety level 2 facilities. Real-time RT-PCR with SARS-CoV-2–specific primers and probes was used to test RNA that was extracted from the samples. A laboratory-confirmed case was diagnosed based on 2-target (open reading frame 1a or 1b, nucleocap-sid protein) positive test by specific real-time RT-PCR. If a cycle threshold value (Ct-value) was less than 37, the result was positive, and a Ct-value of 40 or more was defined as negative. If the Ct-value was 37 to less than 40, retesting was required. A repeated Ct-value, testing less than 40 and observed with an obvious peak, was defined as a positive result.

### Statistical Analysis

The database was established by Excel 2010. SPSS v.18.0 for Windows (IBM Corp., USA) was used for statistical analysis and relevant statistical charts. The epidemiological characteristics and incubation period of clusters were described.

## RESULTS

Eleven clusters of COVID-19 were reported in Qingdao City between January 29 and February 23, 2020. In total, 44 confirmed cases were involved in these clusters, which accounted for 73.33% (44/60) of all confirmed COVID-19 cases.

### Demographic and Epidemiological Characteristics

The 11 clusters were reported in 6 districts, with the largest number in Shinan District (3 clusters), the least number in Laixi District and Pingdu District (each 1 cluster), and the rest in Shibei District, Jimo District, and Huangdao District (each 2 clusters). Among the 44 confirmed cases, the largest number was in Shinan District (18 cases), followed by Huangdao District (9 cases) and Jimo District (6 cases).

**FIGURE 1:**
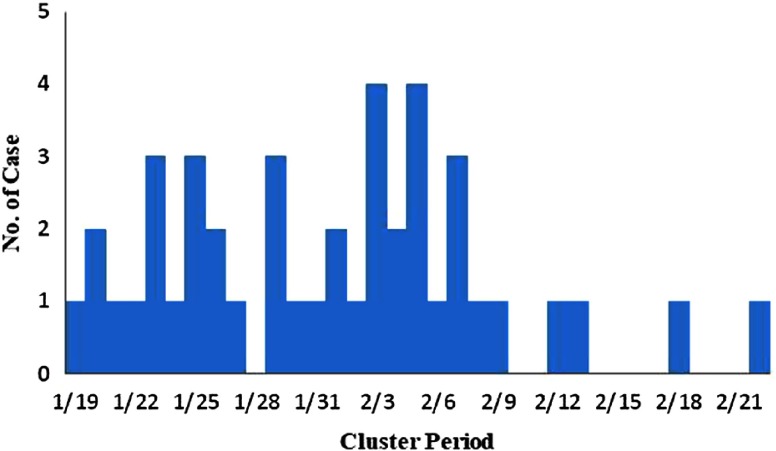
Onset of Illness Among the 44 Confirmed Cases of COVID-19 in 11 Clusters in Qingdao City, China

According to the time of onset, the epidemic of these clusters occurred between January 19 and February 22, 2020, mainly concentrated from January 19 to February 2, 2020, accounting for 84.09% (37/44) of all cases. The largest number of cases occurred on February 3 and 5, both of which were 4 cases (Figure 1).

Patients aged 20-59 y old accounted for the largest proportion (68.18%; 30/44) of cases. Of 44 patients, 15 were male and 29 were female, with a male-to-female sex ratio of 0.52:1. Most patients were employees and retirees, who accounted for 63.64%. Five of the patients were medical staff, 2 of which were infected while traveling and 3 were infected by exposure to confirmed cases (Table 1).

**TABLE 1:** Demographics Characteristics and Clinical Outcomes of 44 Patients in Clusters in Qingdao City

	Patients(*n* = 44)
Age, y, median(SD)	46
Age, y, range	1-90
≤9	3(6.82%)
20-39	14(31.82%)
40-59	16(36.36%)
60-79	9(20.45%)
≥80	2(4.55%)
Sex	
Female	29(65.91%)
Male	15(34.09%)
Occupation	
Retired	8(18.18%)
Unemployed	7(15.91%)
Medical staff	5(11.36%)
Employee	20(45.45%)
Students and children	4(9.09%)
Exposure history	
Imported cases	41(93.18%)
Close contact cases	3(6.82%)
Clinical outcome	
Remained in hospital	1(2.27%)
Discharged	42(95.45%)
Died	1(2.27%)
Signs and symptoms at admission	
Fever	37(84.09%)
Cough	13(29.55%)
Weakness	11(25.00%)
Muscle ache	7(15.91%)
Headache	5(11.36%)
Chest distress	4(9.09%)
Sore throat	4(9.09%)
Diarrhea	1(2.27%)
Clinical test	
White blood cell count(×10^9^ cells per L; normal range, 3.9-9.9)	2(5.41%,↓); 2(5.41%,↑)
Neutrophil count (%; normal range, 50-70%)	6(16.22%,↓); 14(37.84%,↑)
Lymphocyte count (%; normal range, 20-50)	16(43.24%,↓); 2(5.41%,↑)
Lung computed tomography scan positive	33(82.50%)

### Scale, Duration Time of Clusters, and Exposure Person

The number of cases per cluster ranged from 2 to 11 cases (median, 4). There was 1 cluster with more than 10 cases, accounting for 9.09% (1/11); 2 clusters with 5-9 cases, accounting for 18.18% (2/11); and 8 clusters with 2-4 cases, accounting for 72.73% (8/11). Of the 11 clusters, the longest duration time was 15 d, and the shortest was 1 d (Median: 6). The proportion of 6 clusters lasting 1-4 d was the highest (54.55%; 6/11), followed by 3 clusters lasting 5-9 d (27.27%; 3/11) and 2 clusters lasting over 10 d (18.18%; 2/11) (Table 2).

**TABLE 2:** Basic Information on 11 Clusters of COVID-19

	Number of Cases	Population Size (*n*)	Infection Rate (%)	Duration Time (d)
Cluster 1	7	121	5.79	9
Cluster 2	3	8	37.50	8
Cluster 3	3	52	5.77	6
Cluster 4	3	7	42.86	4
Cluster 5	11	188	5.85	15
Cluster 6	3	9	33.33	3
Cluster 7	2	6	33.33	2
Cluster 8	2	26	7.69	2
Cluster 9	2	3	66.67	2
Cluster 10	3	71	4.23	1
Cluster 11	5	92	5.43	5

The cumulative number of exposured persons was 583, with a maximum of 188 and a minimum of 3 (median, 53). The total infection rate was 7.55% (44/583), ranging from 5.43% to 66.67%. As a unit of exposure, the cumulative number of exposured persons was 95 (3-28; median, 11), and the total infection rate was 32.63% (31/95), ranging from 21.43 to 100%) (Table 2).

**FIGURE 2:**
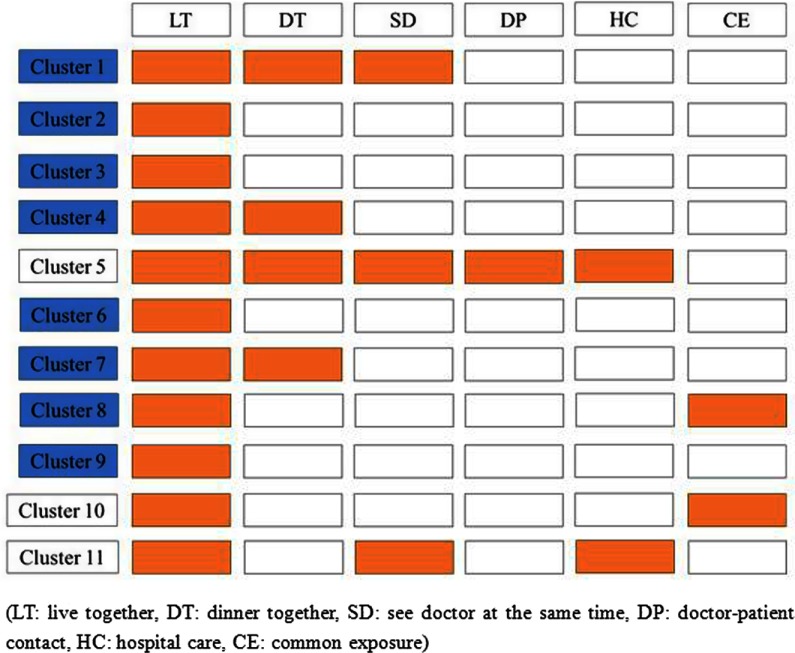
Exposure Mode of 11 Clusters in Qingdao City

In terms of transmissibility, the initial case could lead to a maximum of 10 secondary cases and a minimum of 1 case per cluster (average 4). In terms of exposure mode, family clusters were involved in all 11 clusters. Co-living was the main exposure mode of the 11 clusters (100%; 11/11), followed by dinner together (36.36%; 4/11) and seeing doctor at the same time (27.27%; 3/11) (Figure 2).

### Clinical Manifestation

Of the 11 clusters, the average incubation period was 6.28 d (ranging from 1 to 14 d). On admission, fever and cough were the main clinical symptoms (84.09%, 29.55%, respectively) and diarrhea was found in 1 case. Other symptoms, such as weakness, muscle ache, headache, chest discharge, and sore throat were detected (Table 1).

## DISCUSSION

Recently, the COVID-19 infection has spread rapidly to more than 100 countries.^11-13^ Given that the current COVID-19 outbreak is moving rapidly around the world, reports of the epidemic of clusters for COVID-19 infection are few, which hampers realistic assessment of the coronavirus’s epidemic potential and complicates the outbreak response. We summarize our experiences during these outbreaks to highlight key factors that can help health-care and public health officials prevent family or nosocomial transmission of SARS-CoV-2. In addition, we offer conclusions based on an in-depth, retrospective analysis of the cluster events as they unfolded in these 2 mo.

It was shown that, compared with other districts, more COVID-19 clusters were identified in the area that included the most medical institutions. Nosocomial transmission played a substantial role in initiating and maintaining outbreaks of COVID-19.^14^ In Qingdao, nearly a quarter of the clustered events were detected in Shinan District, where the number of designated hospitals is most. Two typical hospital outbreaks occurred because of iatrogenic infections; furthermore, there is still a risk of recurrence because of the virus’s stealth and complexity. The nursing staff, hospital personnel, and other health-care staff are at increased risk for infection, as observed in the outbreaks. Due to their chaotic management, extensive flow among hospitals, and random activities, nursing staff should be the key population for the prevention and control of hospital clusters; meanwhile, it is necessary to enhance the self-protection awareness and strictly adhere to the use of protective equipment by health-care workers to avoid the occurrence of iatrogenic infection caused by improper personal protection. In addition, the institution of improved triage, limitation of visitors, and fever clinics or designated wards likely led to a decrease in such in-hospital exposures.

Some of the clustered epidemics are characterized by large scale, long duration time, and large number of exposured persons, which makes it more difficult to prevent and control the epidemic. Extremely heavy traffic always occurs during this time period, because plenty of migrants who work in Qingdao go to their hometown before the Spring Festival and, afterward, return back to work,^15^ which will further create favorable conditions for the spread of this difficult-to-control disease and increase the risk of the occurrence of the clustering epidemic.^16^ The considerable high infection rate of the new coronavirus represents a serious threat for the functioning of institutions kept under crowded conditions, such as old-age care institutions, the mental hygiene medical establishment, prisons, and firms with labor-intensive work. Public health authorities might consider active but nonquarantined surveillance in higher-risk settings, such as donning personal protective equipment to prevent contact, droplet, and airborne transmission, enhancing fever surveillance and nucleic acid testing, maintaining a visitor log and limiting visitors, and progressive widening of screening criteria.

Some categories of quarantined close contacts, such as family members, had much higher infection rates than others. The first reported evidence of sustained human-to-human transmission is a family cluster in Shenzhen.^17^ One of the most intriguing aspects of COVID-19 has been the circumstances under which virus is transmitted to large numbers of persons. First, a major social factor influencing disease spread is the arrangement of uninfected contacts between contagious hosts.^18^ Second, it is plausible that closed environments contribute to secondary transmission of COVID-19 and promote super-spreading events.^19^ As a result, the characteristics of relatively closed family space and frequent personal contact increase the risk probability of cluster epidemic in families. IN addition, the high infection rate among family members might reflect that SARS-CoV-2 is highly pathogenic and strongly infectious. Some measures may have contributed to slowing family transmission and bringing the epidemic under control by persuading people to reduce holding family gatherings, educating and motivating the public to protect themselves and fight together to control COVID-19, requiring family members quarantined separately for 2 wk after their last exposure to a COVID-19 patient who had close contact with probable or suspected COVID-19 patients, disinfecting thoroughly the house of confirmed cases until the health department certifies it does not pose any health risk.

In Qingdao, as a cosmopolitan city of more than 10,000 square-kilometers with a population of nearly 10 million in eastern China, we are at risk of clusters of COVID-19 infection. Pooling of information about clusters may help shed additional light on the special set of circumstances required to disseminate infection to large numbers of contacts. The experience learned from controlling these clusters can hopefully serve to inform future responses to COVID-19, if it were to emerge elsewhere in the world.
